# Envisioning social drones in education

**DOI:** 10.3389/frobt.2022.666736

**Published:** 2022-08-25

**Authors:** Wafa Johal, Doğa Gatos, Asim Evren Yantac, Mohammad Obaid

**Affiliations:** ^1^ School of Computer Science and IS, Faculty of Engineering and IT, University of Melbourne, Parkville, VIC, Australia; ^2^ Media and Visual Arts Department, Koç University, Istanbul, Turkey; ^3^ Interaction Design Unit, Department of Computer Science and Engineering, Chalmers University of Technology, Gothenburg, Sweden

**Keywords:** social drone, education, human drone interaction, robot design, remote design workshop, robots in education

## Abstract

Education is one of the major application fields in social Human-Robot Interaction. Several forms of social robots have been explored to engage and assist students in the classroom environment, from full-bodied humanoid robots to tabletop robot companions, but flying robots have been left unexplored in this context. In this paper, we present seven online remote workshops conducted with 20 participants to investigate the application area of Education in the Human-Drone Interaction domain; particularly focusing on what roles a social drone could fulfill in a classroom, how it would interact with students, teachers and its environment, what it could look like, and what would specifically differ from other types of social robots used in education. In the workshops we used online collaboration tools, supported by a sketch artist, to help envision a social drone in a classroom. The results revealed several design implications for the roles and capabilities of a social drone, in addition to promising research directions for the development and design in the novel area of drones in education.

## 1 Introduction

Advances in Human-Robot Interaction (HRI) have recently opened up for the rising research field of Human-Drone Interaction (HDI). The field generally started by investigating novel interaction approaches such as defining visual representations of a drone Szafir et al. (2015), designing ways for motion control [Obaid et al. (2016a); Walker et al. (2018)], exploring social body motions Cauchard et al. (2016), or defining interpersonal spaces Yeh et al. (2017). In parallel, researchers have looked at utilizing drones in several application domains [see Obaid et al. (2020a)], such as entertainment Rubens et al. (2020), sports [Romanowski et al. (2017); Mueller and Muirhead (2015)], domestic companions Karjalainen et al. (2017), local services [Obaid et al. (2015b); Knierim et al. (2018)], videography Chen et al. (2018), art Kim and Landay (2018), and more. A recent review by Baytas et al. (2019) on designing drones, suggests that drone application domains that target domestic-human environments can be defined as “*social drones*”. Based on a more recent HDI survey by Tezza and Andujar (2019), it is foreseeable that drones will become a ubiquitous technology deployed in many new application domains within our society, but they have not yet been investigated. In their survey, it is suggested that one way to move forward is to gauge research efforts into activities that will elicit design implications for the different societal application areas, enabling a better understanding and acceptance when utilizing drones in our society.

**FIGURE 1:**
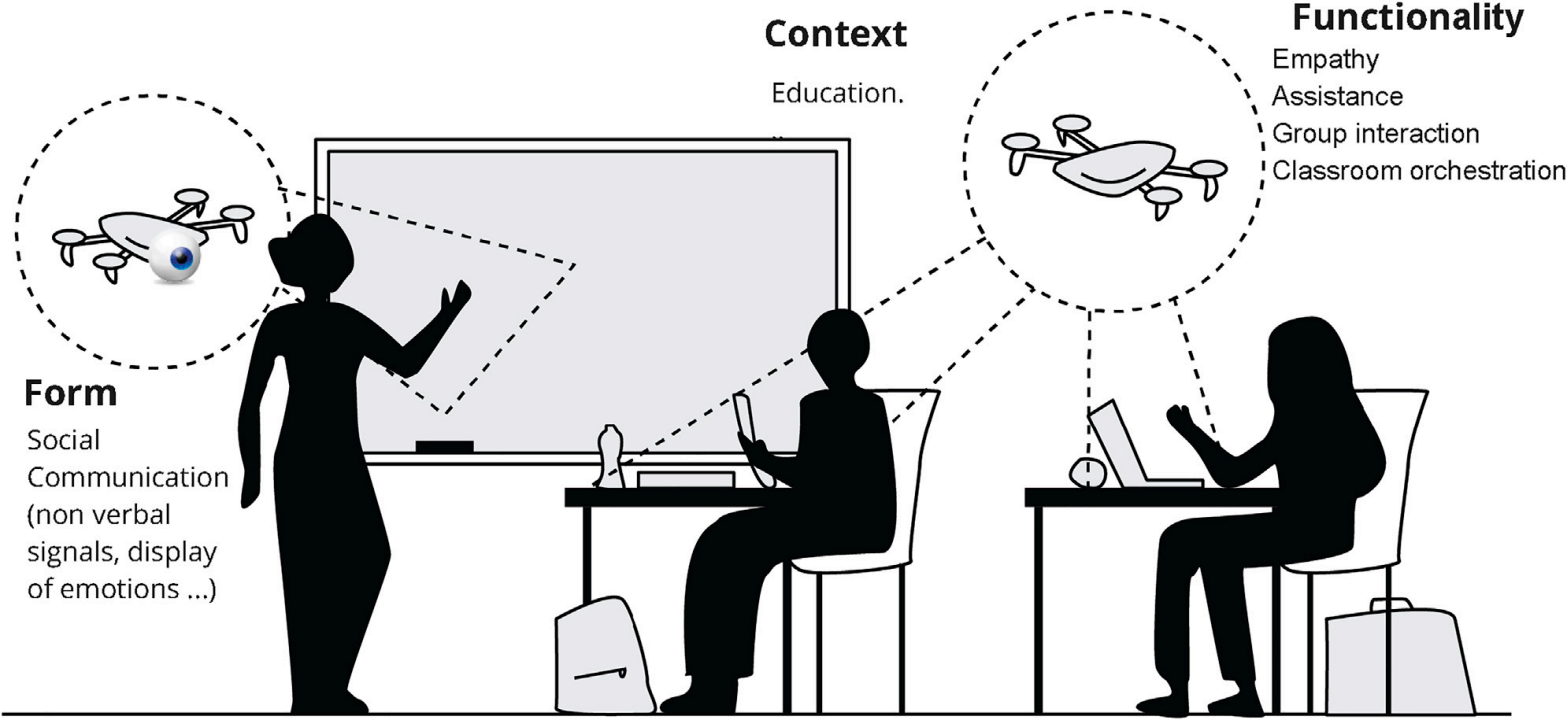
An illustration of social drones in a classroom, with examples of instantiating the social form and social function in an educational context [extending the definition by Hegel et al. (2009)].

Extrapolating from the social HRI field, a large body of research efforts have been put towards the application domain of education Johal (2020). One of the aims is to introduce novel ways to support teachers in classroom environments Belpaeme et al. (2018), thus enhancing the students’ learning capacity. In this context, ongoing HRI research suggests that assistant classroom robots is a preferred approach Ahmad et al. (2016). However, to the best of our knowledge, the application domain of social drones (or flying social robots) in education has not been researched yet. Therefore, we believe this is an opportunity to explore the design space and implications of drones in an educational context, in particular looking at drones that support the classroom environment with no intention of replacing the teacher (see Figure 1). We do this by taking a novel first step into exploring the educational drones’ design space and contributing the following to the HDI community:

• Conduct novel research to understand how social drones can be utilized to support the classroom environment, teachers and students.
• Create a user-centered design method to elicit design implications on four main design themes, thus providing insights on social drones in education and future research directions.
• Identify the implications and lessons to be learned from the online and remote design workshops.

## 2 Related work

In this section, we highlight related research to demonstrate the need to establish a design space for a novel application area of social drones in education. The section is divided into three parts: social robots in education, user-centered robot design, and related work towards our approach to drones in education.

### 2.1 Social robots in education

Since the establishment of the Human-Robot Interaction (HRI) field, a considerable research body has contributed to investigating social robots in education [Mubin et al. (2013); Belpaeme et al. (2018); Johal (2020)]. Initially, educational robotic agents originated from research on virtual agents that aimed at enhancing the learning environment of students [Johnson et al. (2000); Yılmaz and Kılıç-Çakmak (2012)]. Thereafter, the physical embodiment of a robot in a classroom environment has attracted the attention of researchers, strengthening several dimensions in a learning classroom setup [Saerbeck et al. (2010); Köse et al. (2015); Kennedy et al. (2015)]. A recent review by Belpaeme et al. (2018) highlights the benefits of having a physically embodied social robot that has a tutoring role in a classroom. One of the benefits is the ability to foster engagement, creating a positive learning experience for students. In addition, in their review Belpaeme et al. (2018) demonstrated that HRI literature used a wide variety of robot appearances; pointing out that almost all of them had social attributes and features (i.e. humanoid features such as head, eyes, mouth, arms or legs). Moreover, in another review on robots used in education, Mubin et al. (2013) showed that the role of a classroom robot is generally seen as an assistant or a tutor supporting the teacher and students. To this end, the aforementioned literature reviews suggest that social robots in education are likely to be autonomous in their movements and will depict an assistant to a teacher role in a classroom environment.

In the context of social robots in education, many researchers have worked on investigating interactions using different robotic platforms that are already available in the market, such as the popular NAO robot [Johal (2020); Amirova et al. (2021)]. For example, Serholt et al. (2014) deployed a NAO robot to investigate the school children’s response to a robotic tutor compared with a human tutor while giving instructions to accomplish a task. Another example is using the NAO robot Obaid et al. (2018a) to study the development of empathetic robotic capabilities in educational settings. While such studies hold a significant value in the development and understanding of educational robots in classroom environments, most can be considered to be taking a robot (device)-centric approach to understand robots in classroom settings; thus, revealing few insights about the users’ views of a what a robotic agent should entail from a user-centered design (UCD) prospective. In the review by Johal (2020), it is also noted that research into the educational use of social robots in adult higher education has been almost unexplored.

### 2.2 User-centered robot design

Users’ contributions in the design process of a robot can help fast track their acceptance and usefulness Reich-Stiebert et al. (2019b). Recently, several researchers have employed and developed new UCD approaches in the design and development of robots [Barendregt et al. (2020); Reich-Stiebert et al. (2019a); Leong and Johnston (2016); S̆abanovic et al. (2015)]. Focusing on the domestic robots, the work by Lee et al. (2012b) inspired other researchers to involve users in the design of assistant robots in a classroom environment [Obaid et al. (2015a; 2016b; 2018b)]. Although their work was not focused on robots in education, Lee et al. (2012b) suggested an innovative approach to envision a domestic robot by utilizing sketches and drawings along with four main design themes: the look and feel, interaction modalities, social role, and desired tasks. Later, Obaid et al. (2015a) used a similar UCD approach to investigate how adult interaction designers and school children would envision a robot as an assistant to a teacher in a classroom. In their study, 24 interaction design students and 29 children took part in focus group sessions to draw, describe, and discuss the creation of robot designs based on the aforementioned themes suggested by Lee et al. (2012b). Their results revealed interesting insights into the clear differences between the adult interaction design students’ and children’s views. For example, children wanted their robot to have a human-like form that included robotic features, but adult designers envisioned a cute animal-like robot in a classroom space. Thereafter, Obaid et al. (2016b) developed a robotic design toolkit (Robo2Box) aiming to support children’s involvement in the design of their classroom robot. The work presented by Obaid et al. (2018b) suggests that social features were envisioned by children. In addition, some children expressed the preference of having a robot with flying capabilities in a classroom.

### 2.3 Human-drone interaction and social drones

One direction in recent HRI research is increasing activity in investigating flying robots (drones), thus creating a whole sub-field on Human-Drone Interaction (HDI). In a recent HDI survey by Tezza and Andujar (2019), research is currently focusing on (1) exploring ways of HDI communications, (2) identifying suitable interaction modalities with drones, (3) investigating human-drone social behaviours (e.g., proxemics), and (4) introducing novel application areas and use-cases. Generally, the two first items are directed towards investigating novel modalities of interaction between humans and drones and the last items investigate novel application domains for these interactions.

For example, studies ranged from exploring ways to navigate/control a drone using body gestures [Obaid et al. (2016a); Cauchard et al. (2015); Ng and Sharlin (2011)], to utilising visual representations held/projected by drones [Scheible and Funk (2016); Schneegass et al. (2014); Romanowski et al. (2017)].

In the case of social drones, however, there is some specificity that will apply. In this section, we propose to define the characteristic of social drones grounding the definition of social robots and highlighting the specificity of social drones. The literature on the design of social drones was recently reviewed by Baytas et al. (2019). In their review, they gave a summary of three main design categories and twelve design aspects that were identified from related literature. The design aspects were put into perspectives in either Drone Design concerns (lighting and displays, proxemics, sound, appeal, control methods, form, flight) or Human-Centered concerns (ergonomics, intuitive control, perceived social role, tactility perception, and intuitive comprehension). While this review is the first focusing on social drones, the authors only provided a short definition, mainly relying on the context of deployment of the drone “*we submit that an autonomous embodied agent in an inhabited space can be similarly described as social.*” While important, social context alone is insufficient to qualify a drone as social.

In Human-Robot Interaction, Hegel et al. (2009) proposed to define social robots as a composition of robot and social interface, with the social interface referring to all the designed features by which a user judges the robot as having social capabilities being: 1) its form, 2) its functionality, which should be exercised in a socially appropriate manner, and 3) its context of use. This definition of social robots can be further defined thus:

Form: All aspects related to the robot communication: its appearance, its socially expressive capabilities (i.e., facial expression, display of emotions, expressive navigation, other types of non-verbal cues)
Function: All aspects which compute any artificial social behaviour of a social robot: BDI reasoning, joint attention mechanisms, theory of mind, affective recognition, and others
Context: All aspects linked to the knowledge of specific applications domains, in terms of social norms and social roles

While presented distinctively, these aspects are obviously interrelated. For example, social functions and social forms would be aligned to meet a user’s expectations (e.g., one would expect that a robot with a mouth could speak).

**FIGURE 2:**
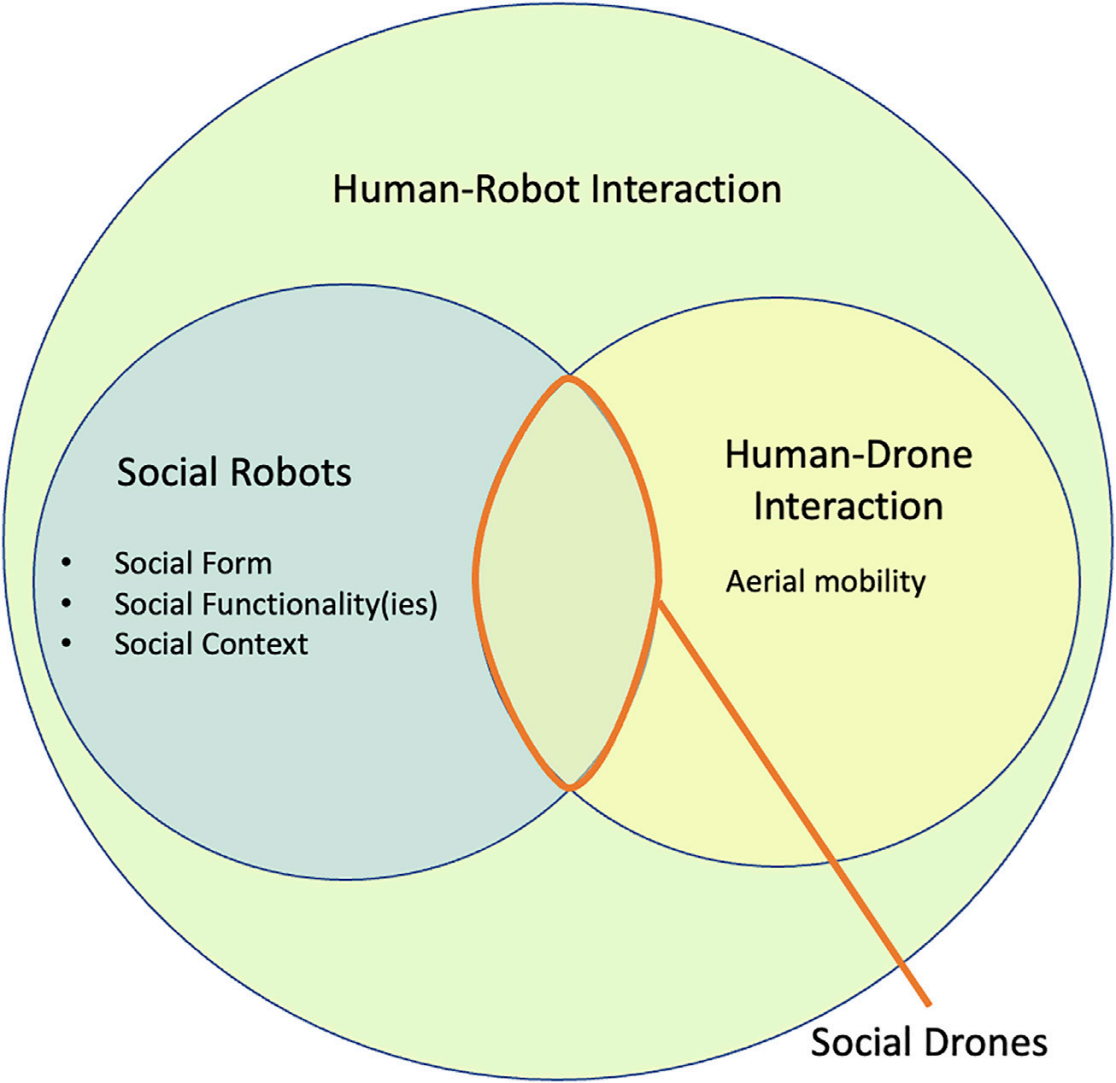
Overview of the research areas linked with social drones.

Going back to the HDI research context, we can now distinguish the social drone as a special case of social robot thanks to its aerial motion capabilities. Looking at the recent literature review by Obaid et al. (2020a), we hence see that researchers are investing these aspects. For example, Herdel et al. (2021) propose to investigate how facial expressions and emotions could be rendered on a face mounted on a social drone (Form). In another example, Karjalainen et al. (2017) investigate how social drones could be used for companionship in the home environment, and what functionality and role these drones would have. Figure 2 summarises the characteristics of social drones in relation to HDI (e.g., interested in interactions between humans in flying robots) and general social robotics.

To summarize, looking at the importance of social robots in education within the HRI field (Section 2.1), and inspired by the domestic companion work of (Karjalainen et al. (2017)) and other related work (Sections 2.2, 2.3), we apply a user-centered method to take the initial steps towards exploring a novel design space on social drones in education. In the following section, we describe our method and the UCD approach in detail.

In particular, we aim to establish how the role envisioned for social drones differs from that of classical social robots in education; what features of the social drones (e.g. aerial motion, bird-view) are envisioned to be useful; and finally, what threats and risks are envisioned in the case of social drones in classrooms.

## 3 Methods

To investigate social drones’ assistance in educational contexts, a set of seven design workshops were conducted remotely, each with 2-4 participants, 2 facilitators, and 1 sketch artist. The study was approved by the institute’s ethics committee and all participants were recruited on a voluntary basis. Their consent was obtained through an online consent form prior to the study. The workshop structure consisted of two steps and the duration of each workshop was 1.5 h on average. A few days after the workshop, a post-workshop questionnaire was sent to participants to evaluate the techniques and tools used (i.e., sketch artist’s support and online tools).

The essence of this study was to take a learner-centered design (LCD) approach, in order to gauge the learners’ environment of an educational drone. We specifically focused on Higher-Education student who are often less researched as a target group for social robots in education (see Johal (2020)).

### 3.1 Participants

An announcement for the study was made via different online channels, including social network accounts and email lists to students at the authors’ institutions. In total 20 participants (15F and 5M) responded to the call and attended the workshops. The workshop announcement was an open call, so there were no criteria for participant selection, however, all the respondents were either university students or graduates (students: three BA, three MA, seven PhD; graduates: two BA, two MA, three PhD). Of the 20 participants eight had teaching experience, while six were specialized in education-related research, four of whom worked particularly on “education for children”. Finally, 10 out of 20 participants had engineering-related backgrounds and 12 out of 20 had design-related backgrounds (two had both backgrounds). One workshop was conducted with four participants, four workshops with three participants, and two workshops with two participants. Table 1 summarises the important demographic information of participants for all the workshop sessions.

**TABLE 1:** Sessions and participants’ information (P#: Participant ID, F: Female, M: Male, D: Design, E: Engineering), All the teaching experience reported was university level.

Session ID	P#	Grad	Gender	Background	Teaching experience
S01	P01	Bachelor	M	E	
	P02	PhD	F	D	Y
S02	P03	PhD	F	D	Y
	P04	Master	F	D	
	P05	PhD	F	D	Y
	P06	PhD	F	D	Y
S03	P07	Master	F	E	
	P08	PhD	F	E	
S04	P09	PhD	F	E	Y
	P10	PhD	M	E & D	Y
	P11	PhD	F	D	Y
S05	P12	PhD	F	D	Y
	P13	Bachelor	F	D	
	P14	PhD	M	E	
S06	P15	Bachelor	F	D	
	P16	Bachelor	F	D	
	P17	Master	M	E	
S07	P18	Master	F	D	
	P19	Bachelor	F	E & D	
	P20	Master	M	E	

### 3.2 Study settings

**FIGURE 3:**
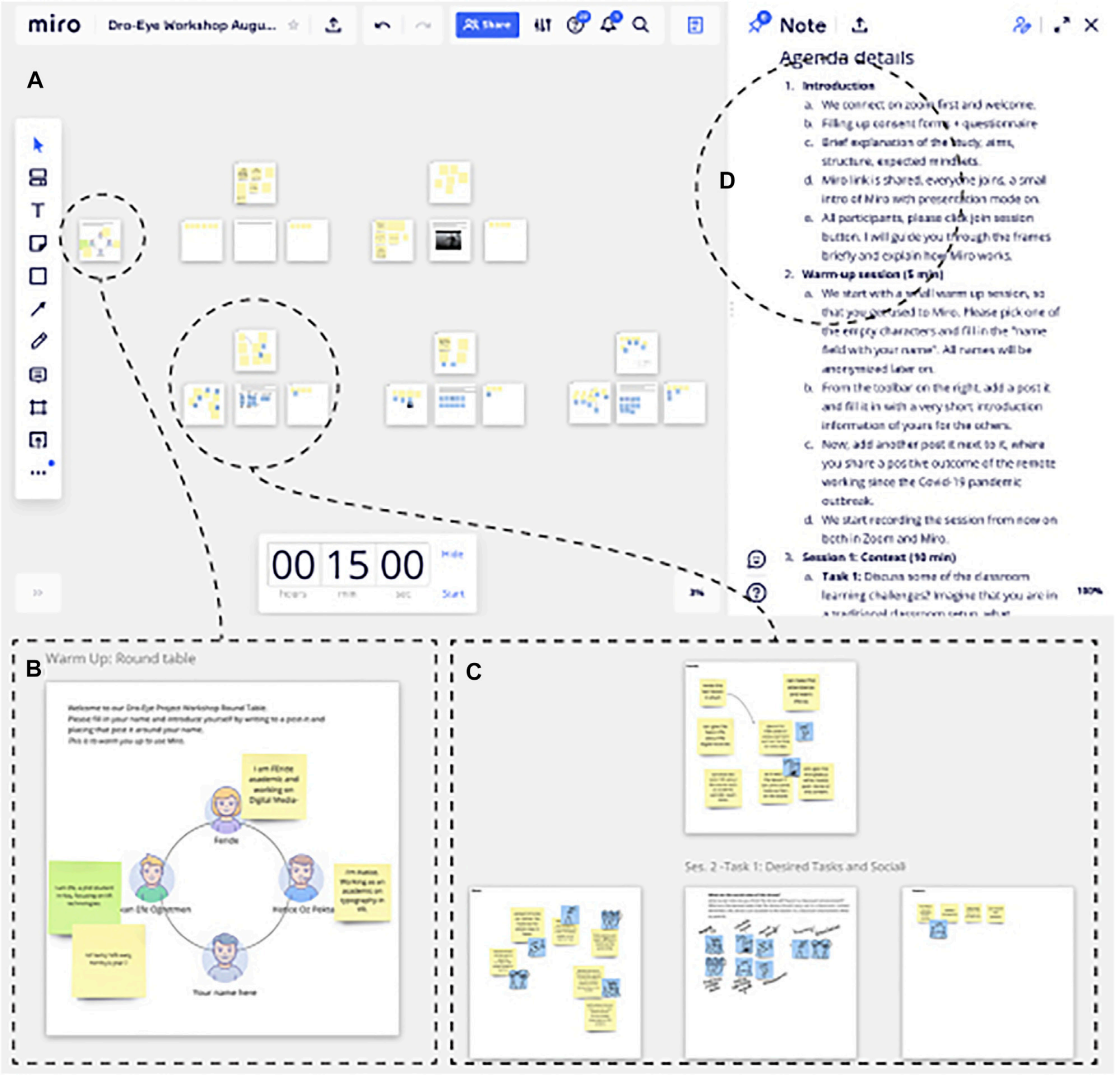
Scene from a Miro session. **(A)** The Miro canvas contained five distinct board groups. **(B)** Orientation board and avatars. **(C)** Each task consisted of one central board and two to four surrounding boards for participants. Each participant had a dedicated seat around the central board, resembling a round-table setting. **(D)** Easy to access workshop agenda for participants’ and facilitator’s reference.

All workshops took place via two online platforms concurrently: Zoom^1^, the video conferencing platform, and Miro^2^. The latter is an online collaboration platform that allows users to share their ideas and comment on shared artifacts on an infinite canvas using sticky notes, drawing tools, and customizable boards (see Figure 3). Besides collaboration, Miro also offers facilitation features such as a timer, an easy-to-access workshop agenda, and directing users’ attention to a specific spot when needed using the visible mouse mode. First, a Miro canvas template was created and used in a pilot study with one participant to evaluate the ease and the flow of the study; thereafter, necessary adjustments were made to revise the workshop structure and the Miro template. The revised template setup was duplicated and used for each workshop. While everyone communicated through Zoom, Miro was used for generating ideas and having discussions on the matter during the workshops. Zoom sessions were audio-recorded, whereas Miro sessions were saved as PDF files and participants’ ideas were exported to a spreadsheet for analysis. One researcher recorded data and managed the technical aspects of the workshop without interacting with the participants, and one researcher undertook the facilitation, guiding the participants through all tasks and managing the discussions as well as keeping track of the time. The reason to use Zoom instead of Miro’s audio communication was to avoid connection problems.

Each session consisted of two steps, each having three tasks, as explained in the below sections. In each task, participants used personal boards assigned to themselves around a central board, resembling a physical round-table setting (see Figure 3). The central board was used by the sketch artist to visualize participants’ ideas while they talked and wrote down their ideas related to each of the tasks. When a task was completed, participants moved to the next round-table to work on the next task. Each participant’s personal board contained three empty sticky notes for them to start each task. They were free to add more but had to at least fill one.

### 3.3 Step one: Context building

The aim of the first step was to familiarize the participants with Miro and the topic of the workshop. It consisted of three tasks:

#### 3.3.1 Task 1: Orientation

Participants were welcomed via an orientation board, where they were asked to choose an avatar, write their name next to it and introduce themselves using sticky notes next to their chosen avatars. After each participant figured out how to use the Miro basic tools needed for the rest of the workshop and had met each other, everyone moved to the next task. For the rest of the workshop, participants had the same position around the board based on the one they picked in this first task, as if they were sitting around a table.

#### 3.3.2 Task 2: Recall a learning challenge

As an introduction to the topic of the study, participants were asked to write down the challenges they face (or had faced) in physical education settings. Each participant presented their sticky notes, and discussed the challenges and issues they had experienced in classrooms. We noticed here that the participants referred mainly to issues encountered in their higher education context.

#### 3.3.3 Task 3: Presentations of current drone applications and capabilities

The general public shapes their conceptual understanding of drones (as a rising technology) from representations exhibited in the mainstream media (e.g., movies); as illustrated by Aydin (2019). This meant that our participants arrived at the study with some previous knowledge of drones from the different media channels. Aydin (2019) also states that the general public is not aware of the majority of applications that drones can hold, giving us an incentive to educate our participants on a broader understanding of the *social drone* space. To achieve this, we used a priming video^3^. After the discussion on the challenges in educational contexts, all participants were briefly introduced to the many current applications of drones by the facilitator, and then watched a YouTube video on social drones created by the MagicLab (2016), illustrating how drones, individually and in groups, could interact with a user, and what their current interaction capabilities are.

### 3.4 Step two: Brainwriting on social drones in education

**FIGURE 4:**
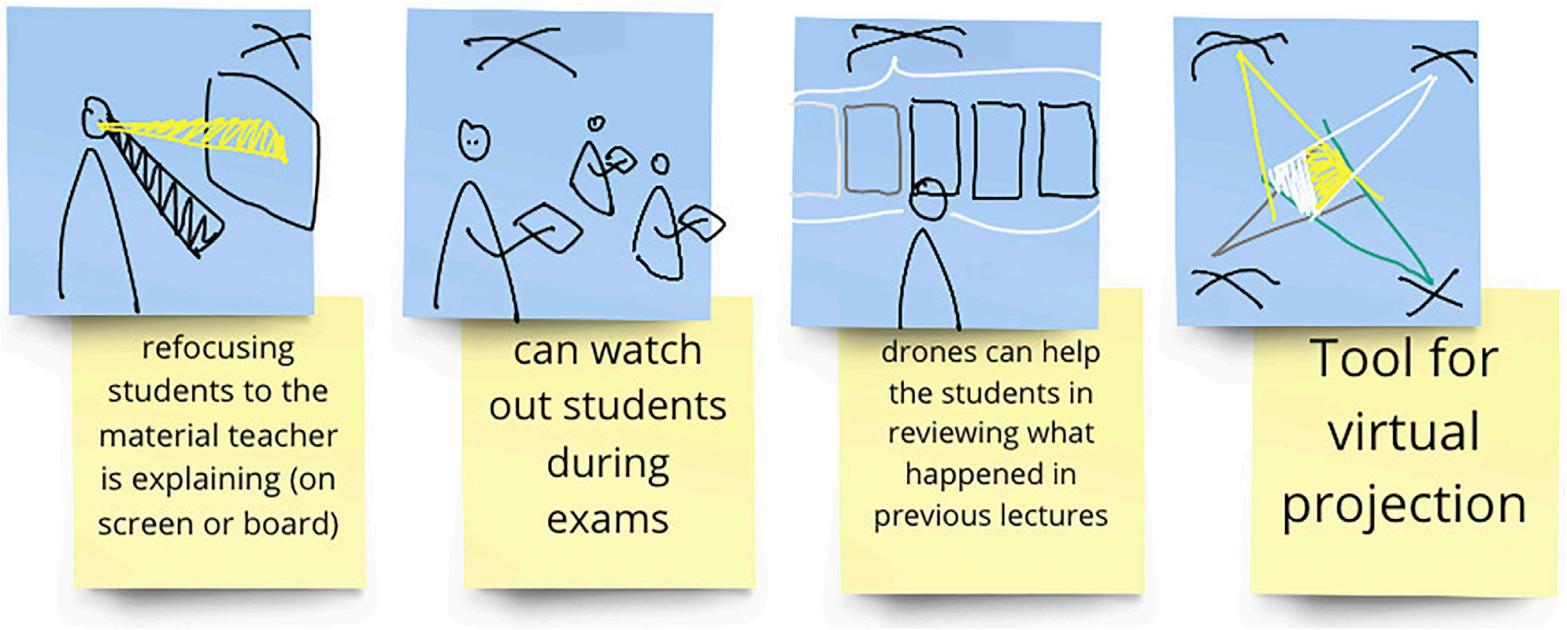
An example of the drawings made by the sketch artist to visualize participants’ written ideas during the fourth workshop. The hovering X shape illustrates a drone. The shape representing the drone was suggested by the sketch artist during the pilot study, to allow the visualization of drones in the quickest way possible.

In this step, the aim was to investigate how participants imagine drones being used in educational settings. Inspired by the work presented by Obaid et al. (2015a), we adopted similar themes to reveal design aspects on social drones in education. Therefore, this step consisted of three theme-based tasks: desired tasks and social roles, interaction modalities, and the look and feel of social drones. Furthermore, for this step, an adapted brainwriting Rohrbach (1969) technique was used, in which participants were asked to individually note down their ideas on sticky notes on their boards before exchanging those ideas in a group discussion. Concurrently with the brainwriting activity, the sketch artist made drawings of participants’ ideas on the central board. Each idea was captured with drawings on separate sticky notes, and those drawings were improved based on the discussion among participants and on their feedback (see Figure 4). Participants were also asked to match the drawings with their own ideas during the discussion; in order to help others to understand and to facilitate the discussion further.

#### 3.4.1 Task 4: Desired tasks and social roles of social drones in education

For this task, the facilitator asked the following questions to the participants and set up a timer for 5 minutes for brainwriting:

• What are the social roles of a drone?
• What social roles do you think the drone will have in a classroom environment?
• What are the desired tasks that the drone should carry out in a classroom context?

Participants were reminded that the drone should be imagined as an assistant supporting a classroom environment, possibly addressing some of the learning challenges they elicited in Step One, Task 2. After the brainwriting activity, the drones’ possible social roles and tasks in educational settings were discussed.

#### 3.4.2 Task 5: Interaction modalities

In this task, the goal was to identify how participants would envision interacting with a drone in an educational setting. They were asked to generate ideas based on the following questions:

• What interaction modalities should this drone have?
• How do you envision your drone interacting with students, with teachers, and with the environment?

The time given for brainwriting in this task was again 5 min.

#### 3.4.3 Task 6: Look and feel

Based on the discussion on previous tasks, the facilitator asked the participants to envision the look and feel of social drones. Within 5 minutes, the participants were asked to think about and note down their ideas on the following questions:

• What do you imagine the drone to look like?
• What would its shape, color, size look like?
• Are there any additional functions or parts that the drone should have that would help with its social role?

### 3.5 Post-workshop questionnaire

A week after each workshop, a questionnaire was sent to the participants to collect their feedback regarding the workshop’s form and content. Each session’s questionnaire started with the pdf export of their workshop discussion boards and participants were asked if they wanted to add anything. The rest of the questionnaire focused on collecting participants’ feedback on the form used to run the workshops (i.e., online tools and the sketch artist’s support). The output of the questionnaire and participant’s feedback are presented and discussed in Section 5.4.

### 3.6 Data collection

All the sticky notes were extracted from Miro and parsed into a table for annotation. The first reading of all the sticky notes allowed us to clean and remove empty ones. The audio recording was also used when the idea written on the sticky notes was not clear. The recording was then transcribed to complement the information on the sticky notes. The analysis process was mainly based on annotating the data logs for the participants from each of the workshop sessions. In this section, we describe the derived coding scheme and the annotated coding process.

### 3.6.1 Coding scheme

We utilised a deductive categorisation approach [see Bengtsson (2016)] to define the coding categories, mainly based on previous literature, the coders’ knowledge, and experience in the HRI field. The coding scheme was built to address our three main research questions. When constructing the coding grid, we referred to the work by Lee et al. (2012b) (i.e., Role, Look and Feel, Desired Tasks and Interaction Modalities), and Yanco and Drury (2004)’s taxonomy to characterize the interaction context (i.e., interaction roles, task type), to define the level of granularity, and Obaid et al. (2015a)’s list of robotic attributes that helped to draft the interaction and look and feel codes (i.e., size, sensors). As a first step, the coders started with the main coding categories adopted from the design themes presented in previous research [Yanco and Drury (2004); Lee et al. (2012b); Obaid et al. (2015a)]. The main themes consisted of the social role, desired tasks, interaction modalities, and look and feel. The coders then started discussing the categories, and iteratively defined coding options together to derive an initial coding scheme. Thereafter, each coder used the initial scheme to annotate a sample data set of 55 sticky notes, allowing the coders to discuss and amend any further code categories to the scheme. The final coding scheme contained the main categories listed in Table 2.

**TABLE 2:** Coding scheme used for the quantitative analysis with possible codes separated by “;” for each category.

Main category	Possible codes
Social roles	Teaching assistant; Butler; Moderator (i.e. between students or between teacher and students); Environment Manager (i.e. classoom facility); Companion; Entertainer; Special Needs Caregiver
Interaction modalities: Social cues input	Gaze tracking; Movement tracking; Face/Object recognition; Video/Image capturing; Speech; Environment sensors; Controller (UI or tangible); Gesture control
Interaction modalities: Social cues output	Gaze; Speech; Non-verbal sound; Lights; Displays; Body color; Body language; Writing or texting; Drawing; Tactile feedback
Drone size (diameter)	Small ( < 20 cm); Medium ( ≥20cm and ≤35cm ); Large (>35cm)
Desired tasks	See Table 3 for details on the codes

### 3.6.2 Coding process

The groups generated various notes during the session. The process started with four coders analysing a whole session, 10% of the data, to deduce an inter-rater reliability check. Each coder separately analysed the data logs of one entire session, composed of 55 sticky notes. Thereafter, an inter-rater reliability was computed via a Fleiss measure (Fleiss (1971)-suitable for more than two raters for categorical ratings). On average, across all of the dimensions coded, the inter-rater reliability resulted in a Fleiss’ kappa of 0.69 (SD = 0.13), which is considered to be a substantial agreement. The coders discussed some of the categories further to eliminate any misunderstanding or ambiguities throughout the process. The annotations of the full dataset then commenced, where each of the coders was assigned a set of coding categories to annotate across all of the seven workshops.

## 4 Analysis and results

This section presents the results of the analysis of the ideas captured by the sticky notes and the sketches across seven workshops (20 participants). All of the participants generated a total of 463 sticky notes. In order to report the results, we propose to articulate them following the design themes proposed by Lee et al. (2012a).

### 4.1 Social roles and desired tasks

Participants (P) were invited to envision what social roles would be desirable for a drone in education to support and assist in a classroom setting. Out of the 498 sticky notes generated during the seven sessions, 100 referred to a social role for the drone in the classroom.

The results show two main roles that were predominantly mentioned by the participants: Teaching Assistant (mentioned 54 times), and Butler (22). Other roles mentioned were Moderator (8), Companion (7) and Environment Manager (6).

While Teacher Assistant/Tutor is quite a commonly found role for social robots in education Belpaeme et al. (2018), the task envisioned for social drones as teacher assistant differs somewhat from that of classical social robots in education.

Furthermore, the role of Butler often cited by our participants has mainly heretofore been in the literature about social robots in a home context Karjalainen et al. (2017) and not educational contexts. The ease of movement of drones could explain this as many butler tasks consist of moving objects around the room.

Table 3 shows the envisioned set of tasks for the social drone. Out of the 498 sticky notes, 122 mentioned a task that could be carried by the social drone. While the set is very varied (we counted 17 different types of tasks), two stand out by the frequency at which they were mentioned by the participants: Managing participation (mentioned 30 times) and *Doing chores* (mentioned 21 times). These tasks are in line with the principal desired social roles discussed before, respectively Teaching Assistant and Butler^4^.

**TABLE 3:** Coding scheme used for the desired tasks for the social drone in classroom.

Task	Occurrence
Managing participation (i.e., answering small questions, grouping students)	34
Doing chores (i.e., cleaning, transferring items, bringing food)	30
Proctoring exams/taking attendance	10
Enriching demonstration and visualization	8
Managing students’ attention/focus	6
Ensuring safety/assisting people in case of emergency	6
Ensuring physical settings and arrangements	6
Ensuring everyone understands, hears the teacher, understands the language	3
Informing students about past/current/future class content	3
Assisting musical/body exercise	2
Recording class/course content/board	2
Enriching teacher’s gestures	1
Broadcasting teacher’s lecture in multiple classes	1
Keeping track of time	1
Helping teachers to evaluate themselves	1
Understanding students’ mood	1

We notice that these tasks differ from classical tasks performed by social drones in other contexts, more often found to be suitable for navigation, well-being, and companionship (Obaid et al. (2020a)). But they also differ from classical tasks attributed to other types of social robots in education which often offer individual assistance Johal (2020) rather than classroom orchestration functions.

In terms of the envisioned target users for the social drones^5^, we notice that the participants mainly mentioned tasks involving individual interactions between the social drone and one student (26 times): “*[It] can help students taking notes when the teacher moved on, it can […] explain any point students missed/didn’t understand*” (P16). They also thought the drones could interact with the whole classroom (11 mentions) by broadcasting information, being used as a tool to do physical demonstrations, assessing and managing the well-being of students at the classroom level: “*Classroom environment can be modified to enable a more productive climate (heat, seating arrangement, etc)*”. Finally, some participants thought the social drone could be useful for group work (6 mentions): “*During teamwork it can guide students or participate as well like a leading team member*” (P16).

### 4.2 Interaction modalities and communication

In *Task 5*, participants were asked to imagine how users could interact with a social drone. We categorised their comments into two types: 1) Social Cues Input-describing how the drone perceives its environment and the users, and 2) Social Cues Output-describing how the drones communicate with the users. The analysis shows that participants often mentioned multiple modalities for the drone to perceive its environment and the users, among which the most mentioned were: Speech/Audio (13), Environment sensors (i.e. temperature, light) (10), Gesture (10), Movement Tracking (9), and Face or Object Tracking (7)^6^. We note here that it is interesting that the participants didn’t think about the fact that the drone’s propeller might be too noisy for microphones to be used. We also note that touch was not envisioned and that the modality of interaction proposed by the participants could be implemented for public space proxemic distance see Mumm and Mutlu (2011).

In terms of social drone expressivity, participants mainly mentioned non-verbal communication cues (only 3 sticky notes mentioned Speech and 2 Non-verbal sound). Body Language was the most mentioned modality (8). For instance, participants thought of using different types of motion to convey messages: “*fast-short movements* vs*. slow continuous movements*” (P12), or to include gestures such as “*nodding*” (P20 and P03). Several participants mentioned lights (4) or body changing colors (3) as a way for the drone to express its mood “*RGB color codes: Green is positive, Red is negative [and] Blue is uncertain*” (P20) or to signal learning phases to the whole classroom: “*The color of the light of the drone might indicate something such as its the time of the lecture for questions and discussion*” (P18). The sound of the drone was often considered to be distracting and only two participants mentioned it as a possible way for the drone to communicate. Three participants also mentioned the possible use of a projector embedded in the drone: “*projection (light, picture)*” (P07). Nearly all the interactions mentioned by the participants were spatially collocated (84), and only three mentioned remote interactions with the drone. This has implications for the drone’s appearance (i.e., in terms of safety and discretion), discussed below.

### 4.3 Look and feel

Nineteen out of the 20 participants mentioned the drone’s size in their sticky notes. To code for the drone size, we decided to split the 19 sticky notes into three categories relative to the size of a regular commercial drone such as the Phantom Dj3 (see Table 2). Analyzing the results, we find that participants mainly mentioned a small hand-sized social drone (10 out of 19 sticky notes). It was often justified by the fact that the drones needed to appear safe and to be discreet. Only two sticky notes mentioned a large drone, anticipating that the drone needed to be big enough to contain and carry objects in the classroom or to arrange the table and chairs after the class.

Related to the drone’s size and shape, three participants mentioned that the drone could change shape as a way to illustrate concepts and enrich presentations:“*take the shape of the Eiffel Tower during a lesson on France*” (P08), or to be transportable: “*folding/unfolding for saving space and being able to use in different sizes*” (P06). Further, on the drone’s shape, three participants suggested that drones should look appealing to children. To that end, cartoon-like, animal-like:“*butterfly*” (P08); “*similar to bird or animal*” (P10); or object-like such as a “*balloon*” (P14) or a “*toy*” (P06) drones were mentioned by several participants. Related to child-friendliness, participants also suggested colorful and easy-to-distinguish drones: “*very colorful like a flying insect*” (P03).

Another aspect related to the look and feel of the drones was the potential distraction that the drones may cause. Seven participants explicitly addressed that the drones should be as silent as possible. Possible solutions suggested for making drones silent were covering or removing the propellers (P19, P10) and using noise-cancelling (P20). One participant mentioned that the drone should be “*silently lurking around*” (P05) so as not to interrupt people in the class. Besides the auditory distractions, five participants were concerned about visual distraction, and suggested the following to render drones less distracting: “*transparent drone*” (P21), “*seamless: change the color according to the environment*” (P11), “*not a vibrant color, plain physical features*” (P10) and “*ghost-like*” (P10).

Non-threatening drones emerged as a need in the classrooms both for children and older students. Participants mentioned drones to be physically harmless and with a friendly appeal. Related to physical safety, soft materials (P11) were suggested. Another suggestion for harmless drones was that “*lights should not be harsh or blinding*” (P20).

In addition to the features related to look and feel explained above, lightweight (P11), color-changing (P10, P02) (depending on e.g., students’ mood (P06, P04), environment (P08), preferred visibility (P12), performed behaviour (P20), social role (P13), activity status (P09) and class subject (P08)), smooth (P03) and modular (P06) drones were suggested.

## 5 Discussion

The main aim of this study is to explore how social drones would be utilised in a classroom setting, in terms of social roles, tasks, interaction modalities, and appearance. Here in this section, we will discuss implications related to the design of social drones in a classroom and the methodological implications associated with the remote design workshops.

### 5.1 Implications for social role

Keeping in mind their learning challenges, several roles were envisioned by participants in the study, but two main roles stood out: a Teaching Assistant (TA) and a Butler. A TA was by far the most mentioned role for the social drone in the classroom. Participants thought of the TA as being able to bridge the communication between the teacher and the students, either by using other media (recording, broadcasting, enriching the demonstrations and visualisations) or through gestures (“*attract the attention of teacher*” [P03]). Similar to a regular classroom TA, the social drones could be asked to gather questions, answer some of them directly, manage group discussion, and balance students’ participation. Low Engagement and lack of focus during the class were discussed to be important challenges by several participants. While several participants thought that the TA could be more accessible and engaging than the teacher, they also expressed concern about the drone being distracting. As a result, the look of the drone incorporates attributes such as small, invisible, and quiet: “*size-not so big, not to distract students so much*” (P16), “*seamless[…]*” (P11) “*transparent drone*” (P21), and “*silent as possible as it can be*” (P10). To summarise, the drone TA is seen as a way to discreetly bridge communication between students and teachers through physical movement.

A Butler was the second most mentioned role, and this is an unexpected role when applied to social robots in education (Belpaeme et al. (2018)). The butler was seen as an assistant in charge of students’ well-being in the classroom, that “*brings food when people are hungry*” (P18), “*handing out/collecting tools, papers*” (P19), “*clean[ing] any left trash[, and] adjusting seats and tables after class is done*” (P01). Because of its ability to easily move in space, the butler drone would be expected to carry objects, and hence was thought of as a larger drone, sometimes with “*extensible arms*” (P01) or a “*small storage (for distributing things)*” (P13). Here also, one aspect participants thought of as important was a safe appearance: “*appear harmless*” (P03), “*it must be made of a soft material due to safety reasons*” (P11), and “*not threatening*” (P20). This new butler role brings new scenario opportunities for social robots in the classroom, closer to what is commonly found in home settings.

### 5.2 Implications for the communication capabilities

The interaction modalities that support the social capabilities of a drone were largely discussed by participants, especially when envisioning how the drone could sense users and its environment. We noticed that participants were more talkative about the social inputs and sensors that the drone could have (55 sticky notes on input sensing capabilities) compared to its ability to express itself (only 28 sticky notes on the output capabilities). This could be a result of the fact that 10 out of 20 participants have an engineering technical background. This has also been seen in previous HRI research, in which interaction with humanoid robots was clearly influenced by the technical or non-technical background of the participants Obaid et al. (2014).

Some participants also thought the drone could be in the classroom to monitor student attendance or exams, hence to “*detect humans*” (P19), or to “*give a warning if it detects suspicious behaviour during exams*” (P13). The well-being of students in the classroom was often discussed with two aspects: the students’ mood (affective state and attention) and environmental factors (temperature and light adjustments). Several participants agreed that the drone could be used to monitor and adapt to a student’s mood: “*Biosensors (Understanding the mood of the user)*” (P02), “*mood detection*” (P18); and to the environment: “*adjust the physical environment for best learning conditions (light, temperature, screen size, etc)*” (P16) and “*[the] classroom environment can be modified to enable a more productive climate (heat, seating arrangement, etc)*” (P05). Social capabilities were attributed to the drone to sense and interact with students similarly to other social robots in education (Belpaeme et al. (2018); Johal (2020)). Additionally, we found that abilities to sense the whole classroom were proposed for social drones by participants which is quite specific to the drone’s ability to adopt a bird’s-eye position and see the entire class (see Section 6.1.4). Having said that, improving social expressivity of social drone is needed as they lack anthropomorphic features used by other social robots in education [e.g., NAO Amirova et al. (2021)].

### 5.3 Implications for the appearance

Some participants thought of the social drone as a *versatile* agent that could change appearance and/or change role: “*modular [drone]*” (P06), “*different colors [could] indicate different social roles (janitor [in] red, helper [in] green, assistants [in] orange etc.)*” (P13), “*changeable to the [curricular] subject and to the environment*” (P08). In addition, two aspects of appearance that predominated the participants’ comments were safety and discretion. These two last aspects are very specific to social drones. Indeed, noise and visual distraction could be a threat for students’ learning. We discuss this aspect further in Section 6.1.1.

### 5.4 Implications for remote design workshops

In this section, we discuss lessons learned from our experience with the online combination of using Zoom, Miro, and sketches employed in a design workshop for drones in education.

#### 5.4.1 Designing online around a virtual table

Miro has provided an online collaboration platform for participants and helped in materializing their thoughts. The 18 participants that responded to the post-workshop questionnaire agreed that it was easy to share their ideas using Miro (4.28 on the 5 point Likert scale). Participants each had their own boards that were positioned as if they were sitting around a table, and when it was time for group discussions, they used the group board in the middle. This setup allowed participants to have individual thinking time when needed and orient joint attention during presentations and discussions. Miro has enabled us to overcome certain challenges encountered in co-located physical workshops, such as getting distracted by others or having difficulties in presenting ideas placed on sticky notes due to the distance among participants. Moreover, this online-remote workshop helped us bring together participants, facilitators, and the sketch artist from all around the world (Turkey, Australia, Sweden and Portugal).

Along with Miro, Zoom was used for the introduction of the workshop, audio communication and recording during the Miro sessions. The decision of leaving the video on or off was left to the participants but it was suggested that they should prioritise their time on the Miro screen to be able to concentrate on the workshop content. Only one participant stated that it would have been better to be able to see others during discussions.

#### 5.4.2 Designing with visual sketching support

The third component of the method we used was the sketch artist’s contribution to the workshops. The idea behind employing a sketch artist was to support participants in presenting and discussing their ideas that would otherwise be written and limited to keywords. That is why the sketch artist simultaneously visualized ideas that participants noted on their boards, and the sketches were placed on the central board while participants explained their ideas in each task. Participants were encouraged to interact with the sketch artist to ask for corrections or comment on the illustrations. Again, the participants mentioned in the post-questionnaire that they were happy with the extent that the sketching artist captured their ideas (4.28 on the 5 points Likert scale). They also thought that the sketches helped them understand the others’ ideas (4.17/5.00); in addition, the sketches helped them enrich their presentations (4.00/5.00). From the perspective of the sketch artist, we realized that it was challenging to visualize multiple participants’ ideas simultaneously, particularly when the number of participants exceeded three. The sketch artist, in the attempt to capture a participant’s idea, also often asked for clarifications in order to be able to draw a corresponding sketch. This was greatly helpful to increase discussions and have participants express their thoughts in more detail.

## 6 Conclusion

In this paper, we have presented a study that aims at envisioning the design of social drones in a classroom context. After running a series of seven online design workshops (20 participants) with the support of a sketch artist, we analyzed, discussed, and extracted several main design implications that can advance future research on social drones in education. To the best of our knowledge, this is the first summary of design implications on social drones in education that contributes to the HDI research and the HRI community in general. We conclude with the following list to shed light on some of the main implications found in our analysis:

1. A teaching assistant and a butler are the main social roles in the context of designing a social drone in educational settings. While the roles that came out from the workshops were relatively similar to other social robots in education [Belpaeme et al. (2018); Johal (2020)]; the findings pertaining to tasks and abilities differed. In particular, we found that the abilities for the drone to easily move in the classroom made participants think of different scenarios: handing sheets of paper, roaming in the classroom to address students’ questions, and acting as a carrier pigeon to carry messages between students. Another main identified role was to be the classroom butler; as discussed in the paper, while common for home robots Lee et al. (2012b), this role has not been explored in the classroom context.
2. A social drone in education should have several interaction modalities to sense the user’s behaviour, particularly the non-verbal behaviours exhibited by the user. In addition, the drone should also have the capabilities to sense its surrounding environment. These are mostly considered as input channels for the drone to make sense of its users and environment and adapt to these.
3. A social drone in education should also be able to exhibit and communicate via different interaction modalities; in particular, the drone should have expressive embodied motions as an output channel.
4. Social Drones could benefit from an adaptive design of their appearance and role. For example, different illuminating body colors could be customised to mean different roles in educational settings.

### 6.1 Research in social drones in education

Currently, Drones in Education can be considered to encompass several realistic scenarios despite the challenges of the technology and the associated infrastructural foundations at educational institutes. For example, drones can be utilized to be used in aerial monitoring of students on the ground, in particular in outdoor settings. Another example, is the use of drones in education as a tool or an applied instrument in educational subjects, such as science and technology. However, envisioning the use of drones as a social entity in education that can deliver and communicate with students has several research challenges that his articles tried to outlined. In this section, we demonstrate some of the challenges and associated research opportunities that entail doing research in the area of Social Drones in Education. Here we outline what HDI researchers can address in their work to help in driving the field towards future scenarios and research in HDI.

#### 6.1.1 Challenge 1: Drones’ noise in the classroom

One major issue with most of the commercial drones at the moment is the noise they make when flying Schäffer et al. (2021). Several mitigations for this issue have been proposed Watkins et al. (2020). The first one is the design of propellers that minimise noise. Other suggestions were to allocate zones and flying path to drones.

##### 6.1.1.1 Opportunity

While this issue remains, it is difficult to envision drones being used during lectures. Researchers and drone designers need to come up with ways to further mitigate for this challenge. For example, can drone’s noise be used as a way to communicate Watkins et al. (2020). Moreover, as suggested by Schäffer et al. (2021), drone noise annoyance might be lower in noisy contexts such as group work or tutorials. However, the main opportunity remains on further work to lower the noise of propellers why in motion.

#### 6.1.2 Challenge 2: Drones lack social cues

Unlike the research literature into social robots for education that often investigate how the robot’s affective capabilities could be used in learning scenarios, the mention of affective cues for social drones was nearly absent in our workshops.

##### 6.1.2.2 Opportunity

The current form of drones is not anthropomorphic, and doesn’t allow it to naturally render emotions. [Duffy (2003) makes the link between anthropomorphism and social capabilities of robots]. In order to render social cues, some previous work investigated how the drone’s flight path could be used see Cauchard et al. (2016) and some very recent work even proposed to embed facial expressions or eyes on flying robots [Herdel et al. (2021); Karjalainen et al. (2017); Treurniet et al. (2019); Obaid et al. (2020b)]. Overall, developing social cues for drones to exhibit and equally perceive is a prominent opportunity for the HDI community to address.

#### 6.1.3 Challenge 2: Drones are scary

The perception of drones as a threat was often mentioned during the workshops and this can be linked with the previous point and to common feelings about drones (Aydin (2019)).

##### 6.1.3.1 Opportunity

During the last part of the workshop (Look and feel of the drone), participants often mentioned friendly and non-threatening look to be an important aspect. More social cues, and “warmer” material could be used to design drones that can more easily be accepted in social contexts. This leads us to further work to be done in the area of drone fabrication for HDI, which we believe is a novel area that is yet to be explored.

#### 6.1.4 Challenge 3: Handling more than one student

In terms of the number of participants, we noticed that compared to the literature on social robots in education (Johal (2020)), there were more mentions of group drone interaction. This can be explained by the fact that drones can take this birds-eye/distanced stance, allowing them to interact with more than just one student at a time. A drone can also easily move in the classroom allowing it to be used as a novel channel of communication between students.

##### 6.1.4.1 Opportunity

While this reduces the opportunities for individualised tutoring scenarios, it opens doors to explore a more school realistic setup in which the ratio would be one drone per classroom. It also open opportunities to expand the current paradigm in social robots for education who tend to be used for personalisation and individualised learning Johal (2020). Finally, a novel direction here can be directed towards research in Drones for Education within the CSCW arena. To the best of our knowledge, very little research has been done there addressing drones in education.

#### 6.1.5 Challenge 4: Novel tasks for the TA

Similarly, the tasks envisaged for the drone were close to the ones found in HDI, surveillance (i.e., during exams) and safety Obaid et al. (2020a). While not often studied as social task, the participants thought of a companion, and a safely agent that could be there to guide and monitor.

##### 6.1.5.1 Opportunity

Here again there will be opportunities to develop further research to provide social cues in this kind of scenario in order to inform students when they are being filmed or to guide them in a safe, private and comfortable manner.

#### 6.1.6 Challenge 5: Social drones for classroom orchestration

Our participants mainly focused on the higher education setup (which is not well explored in the research literature for social robots in education). Several scenarios were described but often the social drone was envisioned to be used in a tutorial/workshop kind of setting rather than individual learning or classroom lecturing. This is an interesting aspect as the classroom orchestration and the teacher cognitive load managing group work needs to be taken into account when introducing technologies for the classroom Shahmoradi et al. (2019).

##### 6.1.6.1 Opportunity

Future works could investigate how to leverage the social drone’s high speed and mobility to use it for classroom orchestration (e.g., classroom monitoring, instructional scripting). An example of this orchestration is the management and monitoring of students’ attention and well-being in the classroom that was often mentioned by participants. In these scenarios, participants thought of the drone as part of an Internet of Things ecosystem in which it could sense and adjust environmental aspects such as the light and the temperature.

### 6.2 Limitations and future directions

The above articulation on the design implications can support several future directions, which are also limitations in the presented study. For example, our focus is to gauge for the learners envisioned drone in education; taking a user (learner) centered design approach. This approach is also supported by others in field, such as the work presented by Reich-Stiebert et al. (2019a) and Lee et al. (2012a). Thus, the recruitment of our participants was done mainly through university student channels, and hence some of the graduate students came with teaching experience. However, we didn’t select or expect our participants to have professional teaching experience, which one could consider as a limitation. In this context, we did notice that participants who had some teaching experience were the ones who thought of some teacher-centric scenarios “*Enables teachers to reflect on their quality of teaching*” (P05). Thus, conducting a study with school children and/or school teachers may further suggest additional valuable insights into the design of social drones in education. Moreover, despite the advantages afforded by our running an online design workshop, developing design insights from a face-to-face focus group workshop may reveal further implications towards social drones in education, as it could allow physical prototyping, such as the work presented by Karjalainen et al. (2017).

In summary, social robots in education are complex and challenging as they involve many stakeholders (i.e., students, teachers, parents, and the educational environment), individual and group interactions, and timely responses. As seen in previous research, drones offer a great potential for social interactions in the various application areas (Obaid et al. (2020a)). Thus, investigating the potential uses of social drones in education allowed us to generate novel perspectives for the design of social drones. We hope this work lays the foundation for the design of novel drones targeting teachers, learners, and the classroom environment.

## Data Availability

The datasets generated and analyzed in this study will be available on request to the corresponding author.

## Footnotes

1
https://zoom.us/

2
https://miro.com

3 Video used for priming: https://youtu.be/B4xtsH6pzoM

4 see the dashboard for the full list: https://apps.streamlitusercontent.com/wafajohal/socialdroneeducation/dev/streamlit_app.py/+/#task-analysis

5 see the dashboard for the full list: https://apps.streamlitusercontent.com/wafajohal/socialdroneeducation/dev/streamlit_app.py/+/#target-user

6 see the dashboard for the full list: https://apps.streamlitusercontent.com/wafajohal/socialdroneeducation/dev/streamlit_app.py/+/#social-cues-input
